# Increasing influenza vaccination coverage in healthcare workers: analysis of an intensified on-site vaccination campaign during the COVID-19 pandemic

**DOI:** 10.1007/s15010-023-02007-w

**Published:** 2023-02-28

**Authors:** Sofie Schumacher, Jon Salmanton-García, Andrea Liekweg, Muriel Rolfes, Danila Seidel, Sibylle C. Mellinghoff, Oliver A. Cornely

**Affiliations:** 1 Department of Anaesthesiology and Intensive Care Medicine, St. Elisabeth Hospital, Cologne, Germany; 2 Faculty of Medicine and University Hospital Cologne, Institute of Translational Research (CECAD), Cologne Excellence Cluster on Cellular Stress Responses in Aging-Associated Diseases (CECAD), University of Cologne, Herderstr. 52, 50931 Cologne, Germany; 3 Faculty of Medicine and University Hospital Cologne, Department I of Internal Medicine, Center for Integrated Oncology Aachen Bonn Cologne Duesseldorf (CIO ABCD) and Excellence Center for Medical Mycology (ECMM), University of Cologne, Cologne, Germany; 4 German Centre for Infection Research (DZIF), Partner Site Bonn-Cologne, Cologne, Germany; 5 Pharmacy Department, University Hospital Cologne, Cologne, Germany; 6 Faculty of Medicine and University Hospital Cologne, Clinical Trials Centre Cologne (ZKS Köln), University of Cologne, Cologne, Germany

**Keywords:** Influenza, Vaccination, Healthcare workers, Vaccination campaign, COVID-19, Vaccination coverage

## Abstract

**Purpose:**

Influenza infections have substantial impact on healthcare institutions. While vaccination is the most effective preventive measure against influenza infection, vaccination coverage in healthcare workers is low. The study investigates the impact of an intensified influenza vaccination campaign in a maximum-care hospital on influenza vaccination coverage in healthcare workers during the COVID-19 pandemic in 2020/21.

**Methods:**

Building on findings from our previously published review Schumacher et al. (Infection 49(3): 387, 2021), an intensified influenza vaccination campaign comprising a mobile vaccination team providing on-site vaccination and vaccination at a recurring central vaccination site in addition to promotional measures was performed and analysed regarding vaccination coverage. A survey querying vaccination motivation was performed. Campaign strategies and vaccination coverage of influenza seasons between 2017/18 and 2019/20 were analysed.

**Results:**

The influenza vaccination campaign 2020/21 led to a significant 2.4-fold increase yielding an overall vaccination coverage of 40% among healthcare workers. A significant increase in vaccination coverage was observed across all professional fields; especially among nurses, a 2.7-fold increase, reaching a vaccination coverage of 48%, was observed. The COVID-19 pandemic positively influenced vaccination decision in 72% of first time ever or first time in over ten years influenza vaccinees. Vaccination coverage during prior vaccination campaigns focusing on educational measures did not exceed 17%.

**Conclusion:**

A mobile vaccination team providing on-site vaccination and vaccinations at a central vaccination site in addition to promotional measures can be implemented to increase influenza vaccination coverage in healthcare workers. Our concept can inform influenza and other vaccination campaigns for healthcare workers.

**Supplementary Information:**

The online version contains supplementary material available at 10.1007/s15010-023-02007-w.

## Introduction

Influenza is caused by seasonal viruses which increase mortality rates worldwide. Annually, 291,243 to 645,832 influenza-associated respiratory deaths are estimated to occur globally [1]. Influenza vaccination is the most effective form of prevention against infection [2]. Although influenza vaccination is recommended for healthcare workers (HCW) by most public health institutions, vaccination coverage (VC) is disconcerting across Europe [3]. Especially, VC in nursing staff is unsatisfactory and has repeatedly been reported to be lower than in physicians [4–9].

Regarding the impact of influenza in hospital settings, the risk of HCW contracting influenza at the workplace [10–12], the risk of nosocomial influenza infections in patients transmitted by HCW [13, 14] and work absenteeism of HCW due to influenza infection must be considered [15].

To increase influenza vaccination coverage in HCW, education and promotion, incentives, organisational strategies, regulatory measures and combinations of the beforementioned strategies can be implemented [16]. Influenza vaccination policies such as declination forms, “vaccinate-or-mask” policies or mandatory vaccination are associated with highest overall VC [16]. Despite being the most effective strategy, regulatory measures regarding influenza vaccination are infeasible in many countries to date, especially in Europe. Therefore, influenza vaccination is recommended yet not mandatory for healthcare workers in most European countries [17]. In these countries, education and promotion, incentives and especially organisational measures need to be developed and strengthened [16].

Among pre-COVID-19 influenza seasons in Germany, the season 2017/18 was considered severe with 1674 laboratory-confirmed influenza-related deaths and excess mortality of 25,100 reported to the German public health institute *Robert Koch Institute* (RKI). During 2018/19, 182,000 laboratory-confirmed influenza cases were reported. Out of these, 40,000 (22%) were hospitalised and 954 laboratory-confirmed influenza-related deaths were recorded [18]. Spanning only eleven weeks, the influenza season 2019/20 was shorter in comparison with the previous seasons [19].

With the ongoing COVID-19 pandemic, the world continuously faces an unprecedented challenge. To alleviate the overall burden of respiratory disease, efforts needed to be taken to increase influenza vaccination coverage in the season 2020/21. Thus, an intensified influenza vaccination campaign for HCW of the University Hospital of Cologne (UHC), Germany, was conducted. The key component constituted a mobile vaccination team (MVT) providing on-site vaccination (OSV). Here, we present the implemented strategy and respective resulting VC. Additionally, the prior influenza campaigns during the seasons of 2017/18 and 2019/20 at UHC were analysed.

## Materials and methods

### Study setting

UHC is a maximum-care university hospital with 1,540 beds. The number of HCW decreased from 14,272 in 2017/18 to 14,224 in 2018/19 and increased to 14,505 in 2019/20. During the season 2020/21, 15,290 HCW were employed at UHC. Reference date is 31st of December during each season.

#### Baseline concept

The concept of the three seasons prior to 2020/21 is considered the baseline concept. During the seasons 2017/18 and 2018/19, the campaigns included educational, promotional and organisational measures. Vaccination was provided by the occupational health department (OH) during opening hours at OH facilities. Walk-in vaccination days were provided two and three times, respectively, per season at a central vaccination site (CVS). Furthermore, strategies included announcement of the campaign via e-mail to all employees, intranet presence, an interview promoting influenza vaccination in the hospital newspaper and promotional materials such as posters, pens, stickers and buttons. A few, mostly off-campus, institutes received on-site vaccination (OSV). During 2019/20, two new concepts were included on top of the before mentioned strategies. Firstly, a single mass vaccination event (MVE) for medical students was established. Secondly, educational measures were increased by distributing postal cards which addressed common misconceptions about influenza vaccination (Supp. 1).

### Intervention

#### Concept of the intensified influenza vaccination campaign 2020/21

An intensified influenza vaccination campaign was conducted between beginning of September and end of December 2020. The concept of the intensified influenza vaccination campaign consisted of organisational as well as educational and promotional elements, the focus being an MVT providing OSV as well as vaccination at a CVS. In addition to these interventions, employees could receive vaccination during the opening hours at the OH as before. The campaign was announced by the hospital board to all employees via e-mail. As a reminder, monthly e-mails were sent to employees providing news of the campaign. Considering the COVID-19 pandemic, all elements of the campaign were conducted in accordance with safety measures required by national public health authorities as well as the UHC hospital board.

An MVT was set up comprising a physician and nurse; both were employed for the conduct of the campaign. OSV was provided on a flexible time schedule often during shift changes or at the beginning or end of recurring team meetings to reach as many employees as possible at the same time. Team members could arrange an appointment for their team via a central email account or phone to receive influenza vaccination on-site at wards or institutions. The MVT reached out via email and phone to all remaining departments inquiring on the need for on-site appointment. Additionally, the MVT was stationed at the CVS twice a week during lunch time, where HCW could drop in to receive vaccination without having to schedule an appointment. The CVS was in the centre of the hospital campus, next to the cafeteria and easily accessible for every employee. All employees were informed that vaccination was taking place whenever a flag depicting the campaign logo was set up outside the CVS. This allowed for the MVT to set up additional CVS vaccination slots spontaneously. At the end of the campaign, three mass vaccination events (MVE) for medical students were organised in cooperation with the student body faculty.

**Fig. 1:**
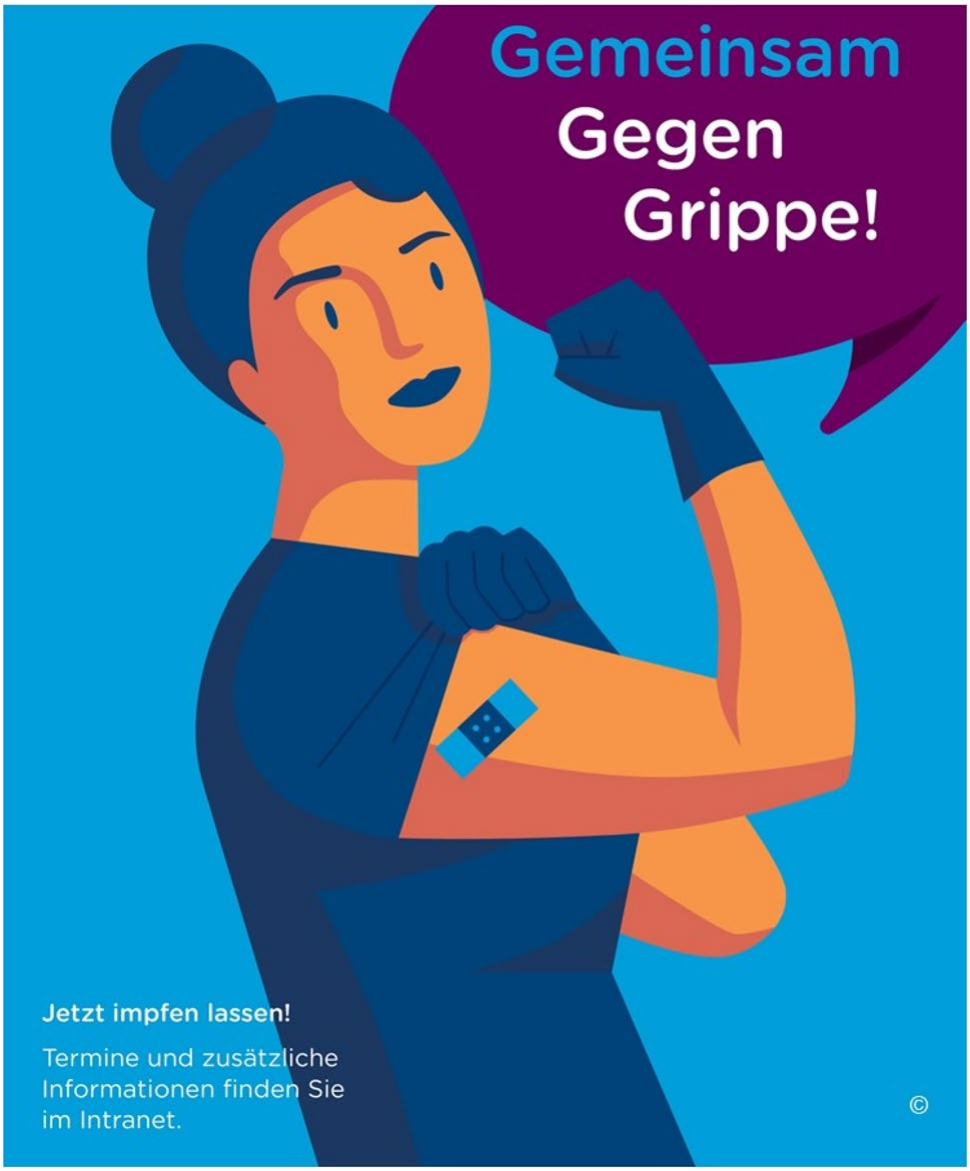
Campaign logo of the intensified influenza vaccination campaign 2020/21. Figure shows the campaign logo of the intensified influenza vaccination campaign in 2020/21 stating “Together against the flu” in the speech bubble and “Get vaccinated now! Dates and additional information can be found on the intranet” in the left bottom corner in German

Furthermore, a promotional campaign was developed. The logo (Fig. 1) showed a vaccinated woman in an empowering pose displaying the vaccinated arm with band aid. This logo was used consistently throughout the campaign. The MVT handed out small packages of hygienic swipes showing the campaign logo as well as stickers and buttons stating “Vaccines save lives” during vaccination appointments. Additionally, two educational videos were prepared. Firstly, an infectious diseases specialist (OAC) employed at UHC addressed concerns of influenza vaccination, such as low efficacy of the vaccine or fear of side effects. Secondly, the department head of nursing appealed to the nursing staff to receive flu vaccination to protect HCW, patients and relatives as well as friends. These videos were circulated via e-mail and shared in the UHC intranet (Supp. 2.). An article about the campaign was included in the autumn issue of the internal hospital magazine at the beginning of the campaign. Furthermore, during the campaign, pictures of vaccinated HCW were taken in the same empowering pose showing their vaccinated arm with band aid. These pictures were available online in the UHC intranet. During the last third of the campaign, a flash mob action of covering bicycle saddles on campus with saddle covers depicting the campaign logo took place.

### Analysis

#### Evaluation of prior influenza seasons 2017/18 to 2019/20

The number of employees receiving vaccination per season with respective age, professional field and sex distribution among vaccinees was provided by the OH department. The information on concepts of the prior seasons as well as the promotional materials was provided by the corporate communications department and infectious diseases division.

#### Data collection during the campaign 2020/21

Due to data protection reasons, the dataset of the season 2020/21 is only complete for HCW vaccinated by the MVT. The age group, sex and professional field of each person vaccinated by the MVT were documented anonymously during vaccination. This information was not documented by those vaccinated at the OH. The complete data set including age, sex and professional field was only available for those vaccinated by the MVT and only the overall number of vaccinated HCW was available for the OH dataset. To estimate the distribution of those vaccinated by the OH per professional field, we performed a web-based anonymised survey (see *Survey on vaccination campaign 2020/21)*. The survey dataset was filtered for the distribution of professional fields among those stating having received vaccination by the OH. The respective distribution of professional fields identified in this survey was extrapolated to the total number of vaccinations performed by the OH. Estimated numbers of those vaccinated by the OH were combined with the data collected by the MVT, resulting in an approximate of the overall VC per professional field for the season 2020/21.

#### Survey on vaccination campaign 2020/21

Following the end of the campaign 2020/21, an anonymous survey was developed to receive feedback on the conduct of the campaign and to complete the unavailable information on vaccinees as mentioned above. The survey was compiled using the EFS Summer 2021 software (TIVIAN, Cologne, Germany). The conduct of the survey was approved by the hospital board, staff committee, data protection officer and equal opportunity commissioner. The survey was e-mailed to all 15,290 HCW once and remained available for three weeks during February and March 2021. The survey queried vaccination status and professional field. If the participating person stated receipt of vaccination, additional questions regarding the motivation to receive vaccination were queried. Also, individuals could give free-text feedback. A translated version of the survey can be found in the Supp. 3.

#### Statistical analysis

Statistical analysis was performed using IBM SPSS Statistics (version 27.0, IBM SPSS Inc, Chicago, IL, USA). Categorical variables such as VC were provided in frequencies, and percentages and proportions were compared using the Chi-squared test. Continues variables were provided by calculating median and interquartile range.

#### Data security

The data collected for analysis during vaccinations by the MVT were collected anonymously. The adherence to data protection guidelines of the data provided by the OH department for the seasons 2017/18 to 2019/20 including the overall number of vaccinations in 2020/21 was confirmed by the UHC data protection officer. Additionally, the UHC data protection office ensured adherence to data protection guidelines of the conducted survey on the season 2020/21.

#### Ethics statement

According to §§ 17 and 40 of the Data Protection Act of North Rhine-Westphalia (Cologne), retrospective analysis and anonymised reporting of patient data without informed consent are appropriate. Further ethical approval is waived.

#### Definitions

HCW in this context refers to any personnel of the hospital and includes UHC employees, medical students of the Medical Faculty of the University of Cologne and subsidiary company employees. Vaccination coverage is defined as the percentage of HCW vaccinated in relation to all HCW. Professional fields were determined beforehand to group vaccinated HCW into a respective category during vaccination by the MVT. These categories included administration staff, functional services, nursing staff, physicians, research personnel, students/trainees/interns and subsidiary company employees.

## Results

### Analysis of past vaccination campaigns

**Fig. 2:**
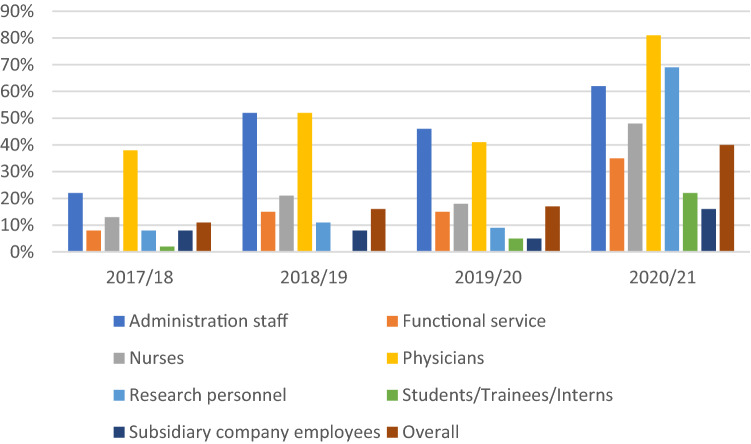
Vaccination coverage overall and per professional field in the seasons 2017/18 to 2020/21. Bar graph shows the vaccination coverage (VC) per professional field as well as the overall VC in healthcare workers per season over the course of 2017/18 to 2020/21. No data were available on the professional field category students/trainees/interns in 2018/19

Following the season 2017/18, a significant increase (*p* < 0.001) in overall VC was observed in the season of 2018/19, yielding a VC of 16% (2289/14,224) in comparison with a VC of 11% (1514/14,272) in the prior season, which corresponds to a 1.5-fold increase. Concerning professional fields, a significant increase in VC occurred in all categories except for subsidiary company employees. Highest relative increase was observed in administration staff (22% (182/819) to 52% (420/809), 2.4-fold increase, *p* < 0.001), followed by functional staff (8% (260/3257) to 15% (472/3187), 1.9-fold increase, *p* < 0.001) and nurses (13% (225/1696) to 21% (354/1668), 1.6-fold increase, *p* < 0.001). Among different professional fields, VC was highest in physicians in the season of 2017/18, further increasing from 38% (538/1422) to 52% (714/1383) in 2018/19 (1.4-fold increase, *p* < 0.001). In the season 2018/19, both physicians and administration staff yielded highest VC reaching 52% (Fig. 2, Table 1).

**Table 1:** Vaccination coverage per professional field between 2017/18 and 2020/21

	2017/18	2018/19			2019/20			2020/21		
	VC in % (Vacc./employees)	VC in % (Vacc./employees)	*p* value (2017/18 vs 2018/19)	Relative change (2017/18 vs. 2018/19)	VC in % (Vacc./employees)	*p* value (2017/18 vs 2018/19)	Relative change (2018/19 vs. 2019/20)	VC in % (Vacc./employees)	*p* value (2017/18 vs 2018/19)	Relative change (2019/20 vs. 2020/21)
Administration staff	22 (182/819)	52 (420/809)	< 0.001	+ 134%	46 (387/836)	0.023	− 11%	62 (542/872)	< 0.001	+ 34%
Functional service	8 (260/3257)	15 (472/3187)	< 0.001	+ 86%	15 (463/3152)	0.892	− 1%	35 (1080/3072)	< 0.001	+ 139%
Nursing staff	13 (225/1696)	21 (354/1668)	< 0.001	+ 60%	18 (340/1920)	0.008	− 17%	48 (1060/2215)	< 0.001	+ 170%
Physicians	38 (538/1422)	52 (714/1383)	< 0.001	+ 36%	41 (599/1457)	< 0.001	− 20%	81 (1215/1505)	< 0.001	+ 96%
Research personnel	8 (76/914)	11 (101/911)	0.045	+ 33%	9 (82/915)	0.131	− 19%	69 (675/981)	< 0.001	+ 668%
Students/trainees/interns	2 (76 /4468)	n.a	−	n.a	5 (235/4421)	−	n.a	22 (1023/4717)	< 0.001	+ 308%
Subsidiary company emp	8 (139/1696)	8 (133/1759)	0.489	− 8%	5 (94/1804)	0.004	− 31%	16 (295/1897)	< 0.001	+ 198%
Other	15	n.a	−	−	n.a	−	−	158	−	−
n. a	3	95	−	−	265	−	−	−	−	−

Table shows the VC per professional field of the seasons 2017/18 to 2020/21 including *p* value of change in VC per professional field between prior and the respective following season calculated using Chi-squared test

*VC* vaccination coverage, *Vacc*. Vaccinations, *n.a.* not available

Between the seasons of 2018/19 and 2019/20 a significant increase (*p* = 0.040), albeit only corresponding to a 1.1-fold increase, in VC occurred as well. The overall VC of 16% (2289/14,224) in 2018/19 increased to 17% (2465/14,505) in the season of 2019/20. Keeping in mind that documentation of professional field of vaccinees was not available for 4% in 2018/19 and for 11% in 2019/20, a decrease in VC per professional field was observed across all fields. Notably, in 2019/20, VC per professional field was highest in administration staff, closely followed by physicians. VC did not exceed 18% in nurses, 15% in functional staff and was lowest in subsidiary company employees (Fig. 2, Table 1).

Demographics of HCW vaccinated over the course of the three seasons between 2017/18 and 2019/20 are shown in Table 2.

**Table 2:** Demographic data of vaccinees during the seasons 2017/18 – 2020/21

	Category	2017/18	%	2018/19	%	*p* value (2017/18 vs 2018/19)	2019/20	%	*p* value (2018/19 vs 2019/20)	2020/21	%	*p* value (2019/20 vs. 2020/21)
Vaccinated/Employees	**− **	**1514**/14272	11%	**2289**/14224	16%	< 0.001	**2465**/ 14505	17%	0.040	**6048**/15290	40%	< 0.001
Age (years) distribution among vaccinated	< 20	14	1%	38	2%	0.056	41	2%	0.993	55	1%	0.003
	20–29	376	25%	656	29%	0.009	777	32%	0.032	1236	20%	< 0.001
	30–39	436	29%	580	25%	0.018	643	26%	0.556	1129	19%	< 0.001
	40–49	293	19%	429	19%	0.638	421	17%	0.135	687	11%	< 0.001
	50–59	295	19%	420	18%	0.380	390	16%	0.021	701	12%	< 0.001
	≥ 60	100	7%	165	7%	0.474	193	8%	0.417	263	4%	< 0.001
	n. a	0	−	1	0%	−	0	−		1977	33%	< 0.001
Sex distribution among vaccinated	Female	904	60%	1380	60%	0.721	1501	61%	0.670	2598	43%	< 0.001
	Male	605	40%	906	40%	0.815	963	39%	0.717	1473	24%	< 0.001
	n. a	5	0%	3	0%	−	1	0%	−	1977	33%	< 0.001
Professional field distribution among vaccinated	Administration staff	182	12%	420	18%	< 0.001	387	16%	0.015	542	9%	< 0.001
	Functional service	260	17%	472	21%	0.008	463	19%	0.111	1080	18%	0.315
	Nursing staff	225	15%	354	15%	0.612	340	14%	0.103	1060	18%	< 0.001
	Physicians	538	36%	714	31%	0.005	599	24%	< 0.001	1215	20%	< 0.001
	Research personnel	76	5%	101	4%	0.384	82	3%	0.052	675	11%	< 0.001
	Students/Trainees/Interns	76	5%	n. a	n. a	−	235	10%	−	1023	17%	< 0.001
	Subsidiary company employees	139	9%	133	6%	< 0.001	94	4%	0.001	295	5%	0.033
	Other	15	1%	n. a	n. a	−	n. a	n. a	−	158	3%	−
	n. a	3	0%	95	4%	−	265	11%	−	−	−	−

Bold indicates the number of vaccinated healthcare workers per season

*n.a.* not available

Table shows the demographic data of vaccinees during the seasons of 2017/18–2019/20. The p-value for the change in distribution among vaccinees in different categories (age, sex and professional field) between the previous and the following season was evaluated using the Chi-squared test. The category “other” applied whenever the professional field was known but did not fit into the given categories

### Analysis of the 2020/21 campaign

The intensified influenza vaccination campaign 2020/21 led to a significant increase in overall VC from 17% (2465/14,505) in the previous season to 40% (6048/15,290) (2.4-fold increase; *p* < 0.001) in 2020/21 (Fig. 2). Excluding medical students, the overall VC increased from 22% (2230/10,084) in 2019/20 to 48% (5025/10,573) in 2020/21 (2.2-fold increase; *p* < 0.001 ).

The increase in VC within each professional field was significant across all professional fields (Table 1). Yielded VC was highest in physicians with 81% (1215/1505, twofold increase; *p* < 0.001), followed by research personnel with 69% (675/981, 7.7-fold increase; *p* < 0.001), administration staff with 62% (542/872, 1.3-fold increase; *p* < 0.001), nurses with 48% (1060/2215, 2.7-fold increase; *p* < 0.001), functional staff with 35% (1080/3072, 2.3-fold increase; *p* < 0.001), medical students/trainees/interns with 22% (1023/4717, 4.4-fold increase; *p* < 0.001) and subsidiary company employees with 16% (295/1897, 3.2-fold increase; *p* < 0.001) (Fig. 2, Table 1).

**Fig. 3:**
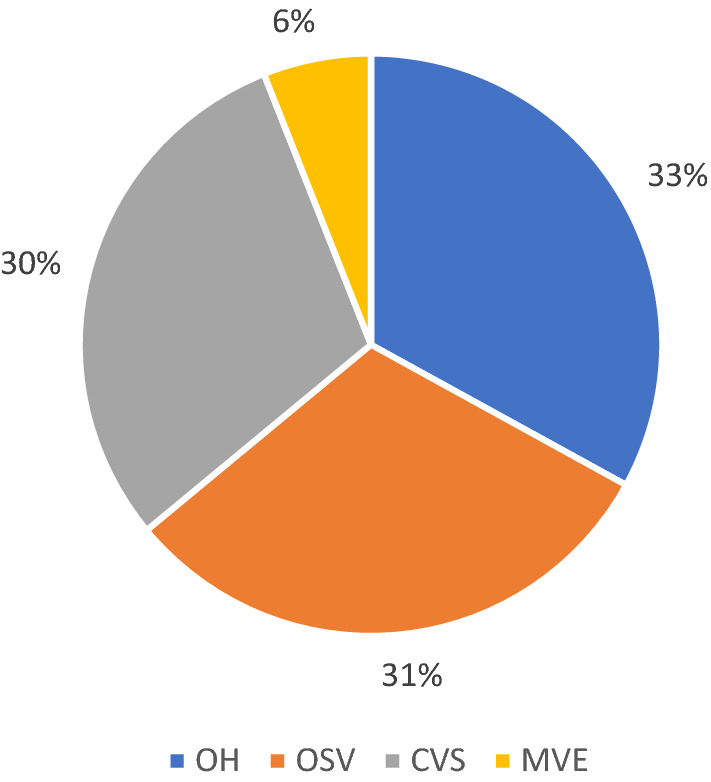
Distribution of vaccinations per provided vaccination site in season 2020/21. *OSV* on-site vaccination, *OH* occupational health department, *CVS* central vaccination site, *MVE* mass vaccination event. Pie chart showing the distribution of vaccinations per available vaccination site or event during the season of 2020/21. Vaccinations at OSV, CVS and during MVE were performed by the mobile vaccination team

Out of all vaccinees, 4071 (67%) were vaccinated by the appointed MVT (OSV, CVS and MVE) while 1977 (33%) HCW received the flu vaccine by the OH. Concerning vaccination sites, 33% received vaccination at the OH, 31% were vaccinated on-site, 30% were vaccinated at the CVS and 6% during MVE (Fig. 3). Out of the 4071 HCW vaccinated by the MVT, 2598 (64%) were female and 1473 (36%) were male, in comparison with the overall sex distribution of 69% female and 31% male employees at UHC. Further demographic information on HCW vaccinated is presented in Table 2.

The demographic data of MVT vaccinees were further analysed regarding the three vaccination sites provided by the MVT. Among those vaccinated by the MVT, the age distribution between OSV and CVS was similar with a median age of 38 (IQR: 29 – 51) at the CVS in comparison with the median age of 37 (IQR: 29 – 49) of OSV vaccinees. Those vaccinated during MVE (almost exclusively medical students) were generally younger (median: 22, IQR: 21 – 24). Regarding sex, 46% of female vaccinees were vaccinated at the CVS, 45% on-site and 9% at the MVE, whereas male vaccinees mainly received vaccination on-site (48%), followed by vaccination at the CVS (42%) and MVE (10%). Physicians and nursing staff both predominantly received vaccination at the CVS (59% and 57%, respectively) in comparison to OSV (41% and 43%, respectively), while research personnel and administration staff predominantly chose OSV (89% and 57%, respectively) compared to vaccination at the CVS (12% and 43%, respectively) (Table 3).

**Table 3:** Demographic data of mobile vaccination team vaccinees of the 2020/21 campaign by vaccination site

VS	Age	Sex		Professional fields							
	Median (IQR)	Female (% per site)	Male (% per site)	Administration staff (% per site)	Functional service (% per site)	Nursing staff (% per site)	Physicians (% per site)	Research personnel (% per site)	Students/trainees/interns (% per site)	Subsidiary company emp. (% per site)	Other (% per site)
CVS	38 (29 – 51)	1203 (46%)	625 (42%)	115 (43%)	473 (52%)	367 (57%)	511 (59%)	53 (12%)	207 (30%)	81 (40%)	19 (95%)
OSV	37 (29 – 49)	1166 (45%)	706 (48%)	154 (57%)	434 (48%)	272 (43%)	362 (41%)	408 (89%)	119 (17%)	122 (60%)	1 (5%)
MVE	22 (21 – 24)	229 (9%)	142 (10%)	0 (0%)	0 (0%)	0 (0%)	0 (0%)	0 (0%)	371 (53%)	0 (0%)	0 (0%)

Table shows the demographic data of MVT vaccinees including age, sex and professional field per vaccination site

*VS* vaccination site, *CVS* central vaccination site, *OSV* on-site vaccination, *MVE* mass vaccination event

**Fig. 4:**
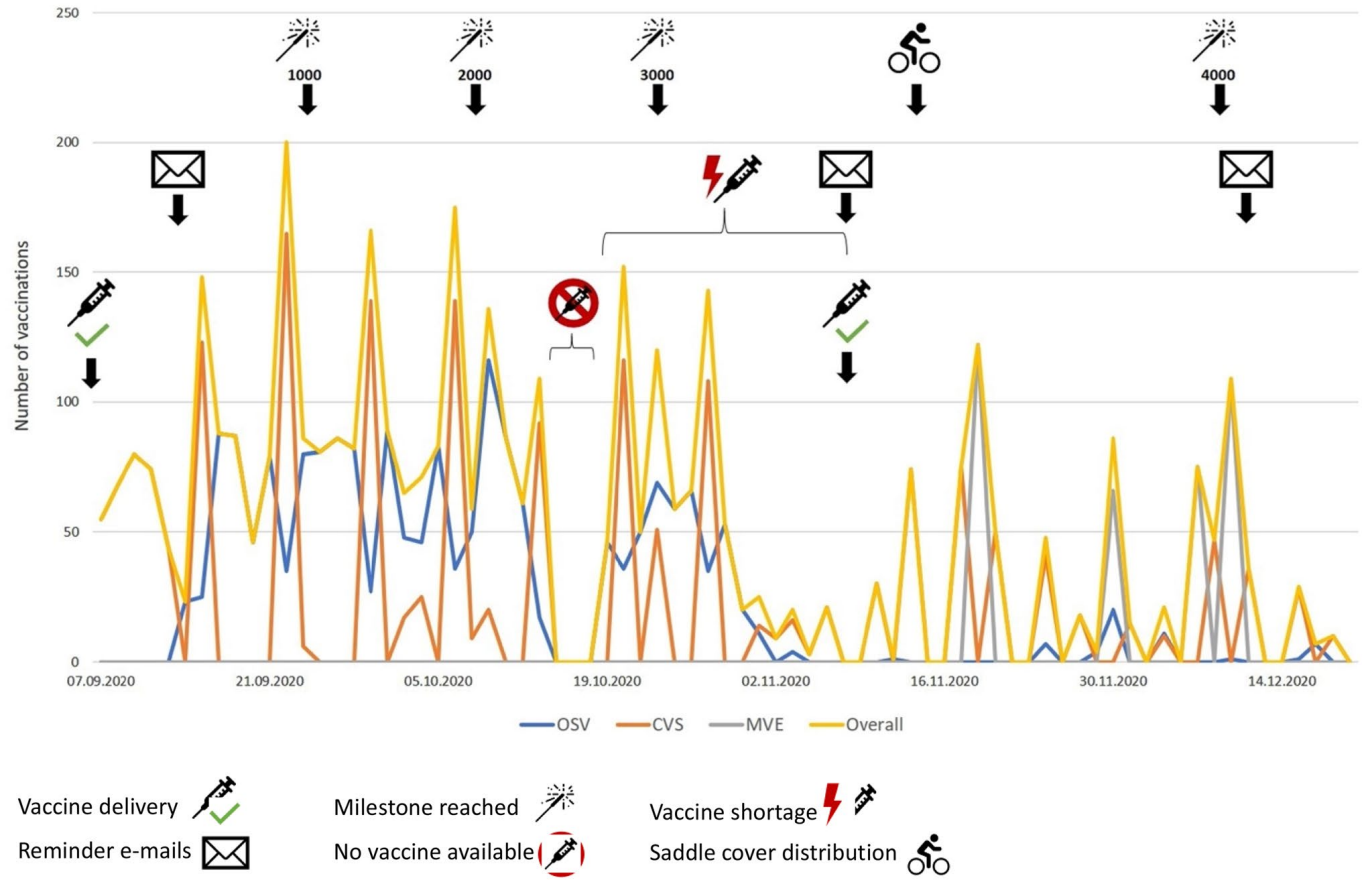
Timeline of the 2020/21 campaign depicting vaccinations per day (conducted by MVT) and corresponding events. *MVT* mobile vaccination team, *OSV* on-site vaccination, *CVS* central vaccination site, *MVE* mass vaccination event. Line graph depicts the number of vaccinations per day conducted by the MVT and corresponding events during the intensified influenza vaccination campaign 2020/21. As the number of vaccinations per day conducted by the occupational health department was not available, only MVT vaccinations per day are shown

The campaign of 2020/21 kicked-off on September 7th, as soon as the vaccine shipment arrived. During the first few days, the MVT was at the CVS daily during lunchtime and HCW could drop in spontaneously. The campaign was officially announced via e-mail to all HCW by the hospital board on September 10th; simultaneously promotional material (posters, pamphlets) was spread across the hospital campus. Almost 3,000 HCW were vaccinated by the MVT by the time a vaccine shortage hit on October 13th which lasted for almost a month. During this time, the opening hours at the CVS were shortened. To inform on the arrival of new vaccine supplies, a second e-mail to all HCW was sent out on November 11th. In the same week, a promotional flash mob of distributing saddle bike covers depicting the campaign logo on bicycles across campus took place. During end of November and beginning of December, four MVE for medical students were conducted by the MVT. Two weeks before the end of the intensified campaign, a third “last call” reminder e-mail was sent out to all HCW. Of note, when comparing Tuesdays (main weekly CVS day) before and after, all three e-mails to HCW by the hospital board led to an increase in vaccinations per day. The milestone of 4,000 vaccinations by the MVT was reached on December 10th. Following the end of the intensified campaign on December 18th, HCW could still receive influenza vaccination at the OH during opening hours in the following weeks (Fig. 4).

### Analysis of the survey following the campaign 2020/21

A response rate of 22% (3327/15,290) was observed in the 2020/21 influenza vaccination campaign survey, which was conducted from February 17th to March 3rd, 2021. Out of all participants, 73% (2413/3327) reported having received influenza vaccination during the 2020/21 season. Out of the reportedly vaccinated employees, 13% (318/2413) stated having received the vaccination externally (e.g. at their primary care physician). Out of all vaccinated respondents, the COVID-19 pandemic contributed to a positive vaccination decision for 30% (720/2413) of those vaccinated. For 40% (968/2413) of those reportedly vaccinated, the intensified 2020/2021 vaccination campaign increased vaccination readiness. For 29% (706/2413), the 2020/21 season’s vaccination was their first influenza vaccination ever or their first influenza vaccination in over 10 years. Out of these first timers, 60% (421/706) stated the intensified campaign had a positive impact on their decision to receive vaccination. Additionally, 72% (510/706) of first timers stated the COVID-19 pandemic positively influenced their decision to receive vaccination. Concerning the response per professional field, for 31% (116/379) of vaccinated nursing staff, the 2020/21 influenza vaccination was reportedly their first flu vaccination ever (or in over 10 years). Out of these first timers among nursing staff, 78% (90/116) stated the COVID-19 pandemic and 53% (61/116) stated the intensified campaign positively influenced their decision to receive flu vaccination. For 43% (50/116), both the COVID-19 pandemic and the intensified campaign contributed positively to their vaccination decision (Table 4).

**Table 4:** Survey results following the 2020/21 campaign by profession

	Overall *N* = 3327 (100%)		Admin. staff *N* = 581 (17%)		Functional services *N* = 300 (9%)		Physicians *N* 412 = (12%)		Research personnel *N* = 449 (14%)		Students/ trainees/ interns *N* = 588 (18%)		Subsidiary company employees *N* = 190 (6%)		Other *N* = 211 (6%)	
	*N*	[%]	*N*	[%]	*N*	[%]	*N*	[%]	*N*	[%]	*N*	[%]	*N*	[%]	*N*	[%]
Vaccinated	2413	73	395	68	214	71	382	93	336	75	430	73	134	71	143	68
Vaccination site																
OSV	729	30	138	35	84	39	107	28	175	52	36	8	53	40	47	33
CVS	682	28	136	34	60	28	144	38	75	22	57	13	42	31	38	27
OH	558	23	77	19	49	23	96	26	60	18	92	21	26	19	39	27
Ex	318	13	44	11	21	10	34	9	26	8	122	28	13	10	19	13
MVE	126	5	0	0	0	0	1	0	0	0	123	29	0	0	0	0
First timers^1^ overall	706	29	124	31	68	32	41	11	126	38	134	31	49	37	48	34
COVID-19^2^ overall	720	30	139	35	82	38	61	16	121	36	104	24	46	34	42	29
COVID-19^2^ among first timers^1^	510	72	98	79	60	88	25	61	91	72	75	56	35	71	36	75
Campaign^3^ overall	968	40	166	42	71	33	126	33	147	44	202	47	62	46	60	42
Campaign^3^ among first timers^1^	421	60	76	61	36	53	24	59	83	66	82	61	30	61	29	60

Table shows the results of the conducted survey on the intensified influenza vaccination campaign 2020/21. The number and percentage of vaccinations as reported are shown for each professional field. Furthermore, the vaccination site per professional field, whether this year’s vaccination was their reportedly first (or first time in over 10 years) vaccination, whether the COVID-19 pandemic positively influenced their decision to receive the vaccine and whether the intensified campaign increased their vaccination readiness in the season of 2020/21 per professional field are outlined

*N* number, *MVT* mobile vaccination team, *OSV* on-site vaccination, *CVS* central vaccination site, *OH* occupational health, *Ex* externally (e.g. primary physician’s office), *MVE* mass vaccination event

^1^First time influenza vaccination ever or in over 10 years

^2^COVID-19 positively influenced vaccination decision

^3^Intensified vaccination campaign positively influenced vaccination decision

The optionally written feedback in the survey repeatedly highlighted the options to receive vaccination spontaneously at the CVS on the way to or from lunch as well as the option to receive OSV. A few comments pointed towards misconceptions, such as not wanting to receive vaccination due to pregnancy and not wanting to receive a live vaccine. Furthermore, reasons for declining vaccination were expressed, such as wanting to be ready for the COVID-19 vaccination at any given moment or believing flu vaccination to be unnecessary due to COVID-19 policies.

## Discussion

The intensified influenza vaccination campaign 2020/21 comprising OSV and vaccination at a recurring CVS led to a significant 2.4-fold increase during the COVID-19 pandemic, yielding a VC of 40% among HCW. Increase in VC was significant across all professional fields. Especially among nurses, a professional field with historically low VC [4–9, 20], we observed a 2.7-fold increase reaching a VC of 48%. Highest overall VC was found for physicians with 81%, while highest relative increase in VC was found for research personnel (7.7-fold). The analysis of pre-COVID-19 influenza vaccination campaigns had underlined the limitations of influenza vaccination campaigns which focus on educational measures instead of exhausting organisational measures, yielding a maximum VC of only 17% [16].

As 67% of all vaccinees were vaccinated by the specifically arranged vaccination team, the appointment of a dedicated team can be considered successful. This corresponds to the findings of an influenza vaccination campaign conducted at another German hospital in 2019 highlighting the importance of a committed team [21]. When comparing the distribution of vaccinees between the three major vaccination sites (OSV, CVS and OH) at our hospital, all three options were equally used. However, keeping in mind that the organisational and logistical time effort is considerably higher for OSV, offering walk-in vaccinations at central sites seems to be more efficient and just as high in demand. Interestingly, nurses and physicians preferably received vaccination at the CVS in comparison to OSV at wards. Furthermore, monthly reminder e-mails increased VC when comparing specific weekdays before and after the notification. The overall VC in the season of 2020/21 may be even higher, as 13% of vaccinated survey participants reported having received vaccination externally. This could be associated with the temporary vaccine shortage during our campaign. Of note, the conduct of the campaign was not compromised during high COVID-19 activity most likely due to strict adherence to COVID-19 safety measures [22].

The survey underlined the impact of the COVID-19 pandemic on the majority of vaccinees, especially among first time (or first time in over ten years) influenza vaccinees. This may point to a change in perception and awareness of vaccinations overall [23–25]. The survey, however, also highlighted the significance of the intensified campaign itself. Interestingly, for first time vaccinated nurses the COVID-19 pandemic seems to have played a major role, positively influencing vaccination decision in 78% in comparison to the intensified campaign itself (53%). Even though educational strategies implemented on their own are not proven to reach sufficient VC [16], misconceptions mentioned during the survey still point to a lack of knowledge even among HCW. Notably, the survey did not query the impact of public German mass media coverage, which during that time strongly highlighted the need for influenza vaccination as well [22].

One other influenza vaccination campaign for HCW during the same period has been published so far [26]. The campaign conducted at an Italian research and teaching hospital comprised educational, promotional and organisational (OSV, CVS) strategies and is comparable to our campaign except for an incentive strategy: a competition ranking VC between hospital departments. Of note, most data were analysed regarding different hospital departments in comparison with professional fields in our analysis. An increase in overall VC from 22% in the previous season to 43% in 2020/21 is comparable to our results. Furthermore, physicians also reached highest overall VC, while highest relative increase was found for administrative staff in comparison to research personnel in our study. While physicians showed a preference for vaccination at CVS in our study, a trend towards OSV was evident at the Italian hospital. While our survey suggests COVID-19 to be a strong motivation to receive vaccination, analysis of a questionnaire on vaccination motivation during the Italian campaign could not show the same trend [26]. However, findings of the 2021/22 campaign at the same Italian hospital underlined the crucial role of COVID-19 on vaccination decision [27].

The analysis of past influenza vaccination campaigns showed a significant increase between all seasons, higher relative change being observed from 2017/18 to 2018/19. This change could be due to the severity of the 2017/18 influenza season in Germany [18]. Even though efforts were taken to increase VC further through educational measures, overall VC did not exceed 17% during 2019/20. This moderate increase in VC is comparable to other campaigns focusing on educational measures [5, 28].

Limitations include the incomplete data set of OH vaccinees due to data protection reasons. Since the professional field of HCW vaccinated by the MVT was categorised subjectively and the distribution of professional fields among OH vaccinees was extrapolated using the survey data, a bias is possible. Furthermore, data collected during campaign and survey were anonymised, precluding a cross-analysis between both data sets.

Despite our endeavours, the WHO-recommended HCW vaccine rate of 75% was not reached [29]. This finding is in line with other pre-COVID-19 influenza vaccination campaigns focusing on educational, promotional and organisational campaign strategies without implementation of vaccine policies [5, 8, 16, 30, 31]. Comparably, VC exceeding 75% among HCW was reached by non-European influenza vaccination campaigns which include policies such as mandatory declination forms, a “vaccinate-or-mask” policy or mandatory vaccination for HCW [16, 32–36]. Other vaccinations, such as measles-mumps-rubella, are mandatory for HCW in some European countries, and the need for such mandates is debatable [17]. In the context of COVID-19, vaccination mandates have gained traction and were realised for HCW in France and Germany [37, 38]. Comparably, COVID-19 VC at our hospital UHC surpassed 90%. An analysis following the implementation of a COVID-19 vaccination mandate for HCW in Italy suggests an increase in vaccine uptake lowering COVID-19 infection rates among HCW [39]. Potentially, COVID-19 vaccine mandates across Europe could pave the way for future influenza vaccine mandates for HCW such as possible influenza “vaccinate-or-mask” policies [36, 40, 41].

Concerning future influenza vaccination campaigns for HCW, a dedicated vaccination team, OSV and recurring vaccination options at a CVS should be integrated [16]. Potentially, a live calendar showing the location of the MVT during OSV in the hospital intranet could help inform on vaccination opportunities and decrease the communication work of the MVT itself. An online tool for appointment scheduling may further streamline the organisation. Furthermore, a decentralised vaccination supply including the distribution of “flu-kits” and “peer-to-peer” vaccination could be implemented [33, 35]. Also, an efficient “blitz” campaign focusing on a shorter time span with a larger vaccination team could be explored [34].

In conclusion, an intensified campaign comprising a dedicated team providing OSV and vaccinations at a CVS in combination with intensified promotional strategies showed a substantial increase in VC in a university hospital setting across all professional fields. The presented concept has potential to be successfully used for upcoming influenza as well as other, including COVID-19, vaccination campaigns for HCW.

## Supplementary Information

Below is the link to the electronic supplementary material.

Supplementary file1 (DOCX 341 KB)

## Data Availability

Data and materials are available upon reasonable request in accordance with General Data Protection Regulation.
