# Supplementary material for: Increasing influenza vaccination coverage in healthcare workers: analysis of an intensified on-site vaccination campaign during the COVID-19 pandemic
Source: Infection. 2023 Feb 28;51(5):1417–29. doi: 10.1007/s15010-023-02007-w (PMC9972307; doi:10.1007/s15010-023-02007-w)
Supplement: Supplementary file 1 — Supplementary file1 (DOCX 341 KB) [file 15010_2023_2007_MOESM1_ESM.docx]

**Supplement 1. Educational postcards addressing misconceptions distributed during the season of 2019/20**

A


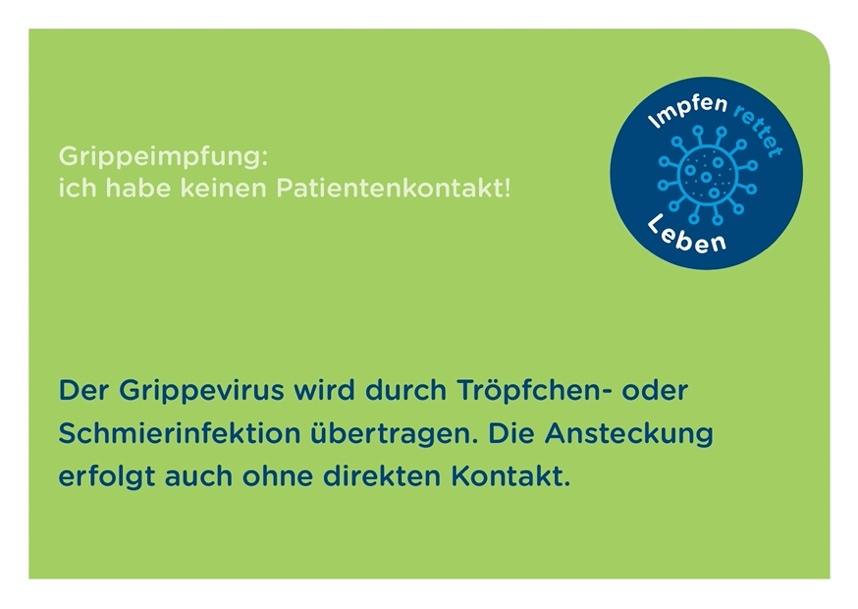


B


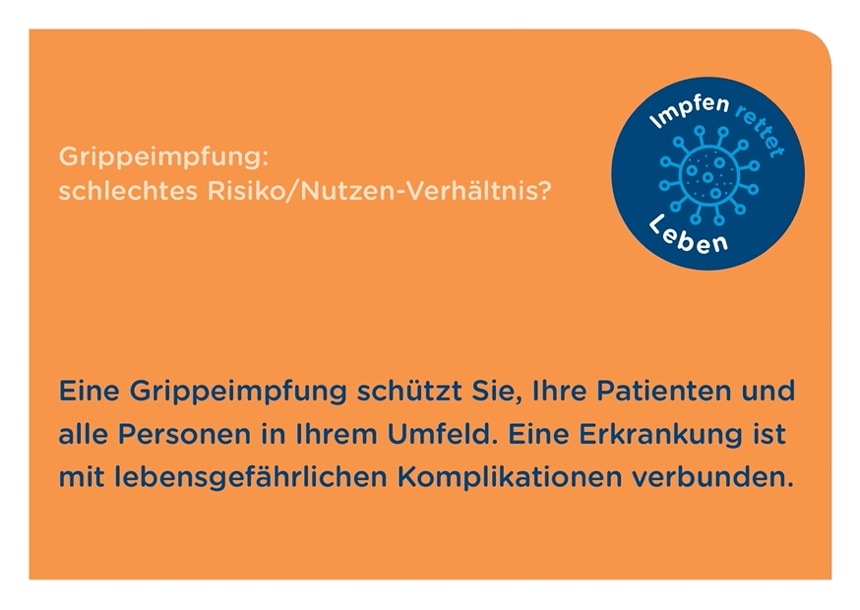


C


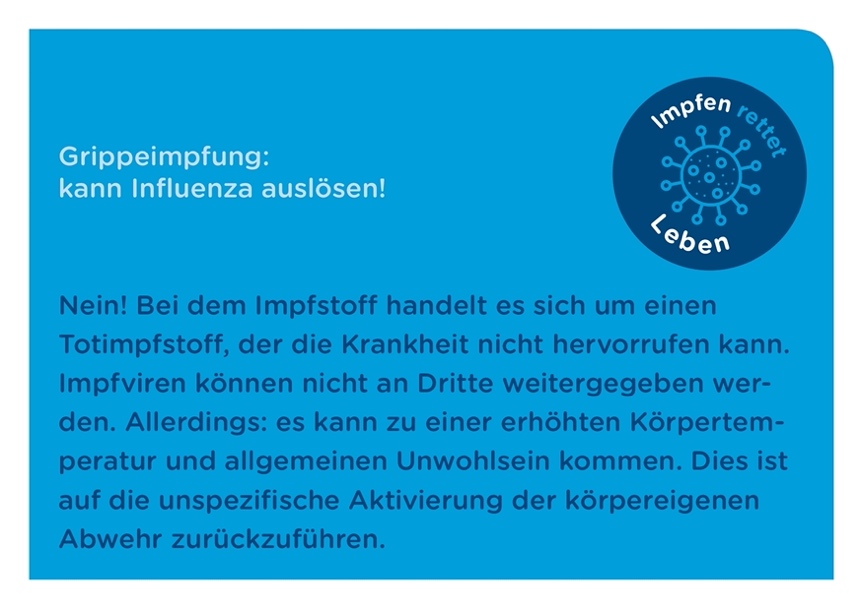


A. Postcard stating “Flu vaccination: I do not have any patient contact! The influenza virus is transmitted via droplets and smear. Transmission is possible without direct contact.”

B. Postcard stating “Flu vaccination: Unsatisfactory risk-/benefit ratio? Influenza vaccination provides protection for yourself, your patients, and others around you.”

C. Postcard stating “Flu vaccination: Can cause influenza! No! The implemented vaccine is not a live vaccine and therefore cannot cause nor transmit influenza. However: Elevated body temperature and malaise is possible following vaccination. This is due to the unspecific activation of the immune system.”

**Supplement 2. Links to educational videos distributed during the intensified influenza vaccination campaign 2020/21**

<https://www.youtube.com/watch?v=Y1Ozg8v16QM&t=13s>

<https://www.youtube.com/watch?v=vBzXNM_8TFs&t=3s>

**Supplement 3. Survey on campaign 2020/21 (translated from German)**

“Introductory text to survey:

Welcome to the survey on the “influenza vaccination campaign 2020/21”

Your participation will support the evaluation of this year's influenza vaccination campaign and help optimize future vaccination campaigns.

Survey:

1. Have you been vaccinated against influenza („the flu“) in the season of 2020/21?
   1. Yes
   2. No
2. Which professional group do you belong to?
   1. Physicians
   2. Nursing staff
   3. Research staff
   4. Administration staff
   5. Functional service
   6. Students/Trainees/Interns
   7. Subsidiary company employees
   8. Other

If yes to 1:

1. Where did you receive the influenza vaccination?
   1. On-site through the mobile vaccination team of UHC
   2. At the central vaccination site
   3. At the occupational health department
   4. I received the flu vaccine externally (e.g., primary health physician) or in a different not here mentioned way

If yes to 1:

1. Did you get vaccinated against influenza for the first time this year or was your last vaccination more than 10 years ago?
   1. Yes
   2. No

If yes to 1:

1. Was the COVID-19 pandemic a decisive factor in your decision to get vaccinated against influenza this year?
   1. Yes
   2. No

If yes to 1:

1. Has the intensified 2020/21 flu vaccination campaign increased your willingness to get the flu shot?
   1. Yes
   2. No

Your comments on the 2020/21 influenza vaccination campaign:

(Text field)

Thanks for the participation:

Thank you very much for participating in our survey
